# Symptom and comorbidity burden in hypertensive patients with obstructive sleep apnea

**DOI:** 10.3389/fendo.2024.1361466

**Published:** 2024-03-04

**Authors:** MengShi Tao, Xiaoqi Dong, Jinjing Tu, Qing Fang, Chuan Shao

**Affiliations:** ^1^Department of Respiratory and Critical Care Medicine, The Affiliated Lihuili Hospital of Ningbo University, Ningbo, China; ^2^Health Science Center, Ningbo University, Ningbo, China

**Keywords:** obstructive sleep apnea, hypertension, daytime sleepiness, nocturia, comorbidity

## Abstract

**Background:**

Obstructive sleep apnea (OSA) is an important but frequently overlooked risk factor for hypertension (HTN). The prevalence of hypertension is high in patients with OSA, but the differences in clinical symptoms and comorbidities between patients with OSA with hypertension and those with normal blood pressure have not been fully defined.

**Methods:**

This study retrospectively analyzed OSA patients diagnosed for the first time in Lihuili Hospital Affiliated to Ningbo University from 2016 to 2020. Patients were divided into an OSA group with hypertension and an OSA group without hypertension. The sociodemographic information, clinical symptoms, comorbidities, and polysomnography results of the two groups were compared. The independent risk factors associated with hypertension in patients with OSA were explored.

**Results:**

A total of 1108 patients with OSA initially diagnosed were included in the study, including 387 with hypertension and 721 without. Compared with OSA patients without hypertension, OSA patients with hypertension were older; had a higher body mass index (BMI) and Epworth sleepiness score (ESS); a higher incidence of nocturia; and a higher proportion of diabetes mellitus, coronary heart disease, and cerebrovascular disease. Multivariate analysis showed age (odds ratio [OR]:1.06, 95% confidence interval [CI]:1.04-1.08), BMI (OR:1.17, 95% CI:1.11-1.23), ESS score (OR:0.97, 95%CI: 0.94-1.00) and nocturia symptoms (OR:1.64, 95% CI:1.19-2.27) was independently associated with hypertension in OSA patients, and comorbid diabetes (OR: 3.86, 95% CI: 2.31-6.45), coronary heart disease (OR: 1.90, 95% CI:1.15-3.16), and ischemic stroke (OR: 3.69,95% CI:1.31-10.40) was independently associated with hypertension in OSA patients.

**Conclusion:**

Compared to OSA patients with normal blood pressure, OSA patients with hypertension had more significant daytime sleepiness, more frequent nocturnal urination, and a higher risk of diabetes, coronary heart disease, and cerebrovascular disease.

## 1 Introduction

Obstructive sleep apnea (OSA) is the most common sleep-related breathing disorder and is characterized by recurrent complete or partial collapse of the upper airway at night, leading sleep fragmentation and frequent nocturnal hypoxia. Approximately 936 million adults aged 30-69 suffer from OSA worldwide; China has the largest number of OSA patients in the world, with a prevalence of 23.6% in people aged 30-69 (1). As the population ages and the prevalence of obesity increases, the prevalence of OSA is also likely to increase rapidly. OSA can cause a series of pathophysiological results such as chronic intermittent hypoxia, oxidative stress and inflammatory response, increased negative pressure in the pleural cavity, and increased sympathetic nerve activity (2). Patients with OSA may present with a variety of symptoms, including habitual snoring, fatigue, poor sleep quality, excessive daytime sleepiness (EDS), memory loss, headaches, and psychological disturbances (3). Many studies have confirmed that untreated OSA can damage multiple systems and organs and cause a variety of complications, including cardiovascular diseases, ischemic stroke, metabolic-related diseases, and neuropsychiatric complications (4–7).

OSA and hypertension are closely associated. The prevalence of OSA in hypertensive patients is approximately 50%, the prevalence of OSA in patients with resistant hypertension is up to 71%, and the incidence of hypertensive crisis in OSA patients with hypertension is 15.70% (8). Approximately 30 to 50% of patients with OSA also have hypertension, which increases the risk of cardiovascular disease and cardiovascular death in these patients (9). Kario et al. found that OSA-related hypertension has the following features, which is usually characterized by increased diastolic blood pressure and nocturnal hypertension typically presents with nocturnal blood pressure may be non-descending (a small or absent nocturnal blood pressure drop) or ascending (nocturnal blood pressure higher than daytime blood pressure) (10). Cai et al. found that the incidence of occult hypertension in patients with OSA was higher than that in those without OSA, and the incidence of occult hypertension in subjects newly diagnosed with OSA was close to 30% (11). OSA induces intermittent hypoxia, which causes oxidative stress and inflammation, leading to vascular endothelial dysfunction, reduces the availability of nitric oxide and vascular endothelial relaxation ability, upregulates sympathetic nerve excitation, and causes vasoconstriction, increased vascular resistance, and vascular remodeling. Hypoxia leads to the activation of the renin-angiotensin-aldosterone system (RAAS), which in turn leads to hypertension (12–16).

To date, the clinical characteristics of patients with OSA and hypertension have not been fully clarified, and only individual studies have been conducted in Western countries. Moreover, OSA is a heterogeneous disease with some racial differences. Craniofacial structure differences have been proposed as one of the important reasons for the greater susceptibility of Asians to OSA (17). Besides, ethnic differences in body fat distribution, the impact of BMI on AHI and genetic susceptibility may be other factors of OSA characteristics in Asian populations (18, 19). This study examined the association of coexisting hypertension with OSA-related symptoms, other comorbidities, and polysomnography (PSG) outcomes in Chinese patients with OSA at first diagnosis.

## 2 Materials and methods

### 2.1 Study participants

**Figure 1:**
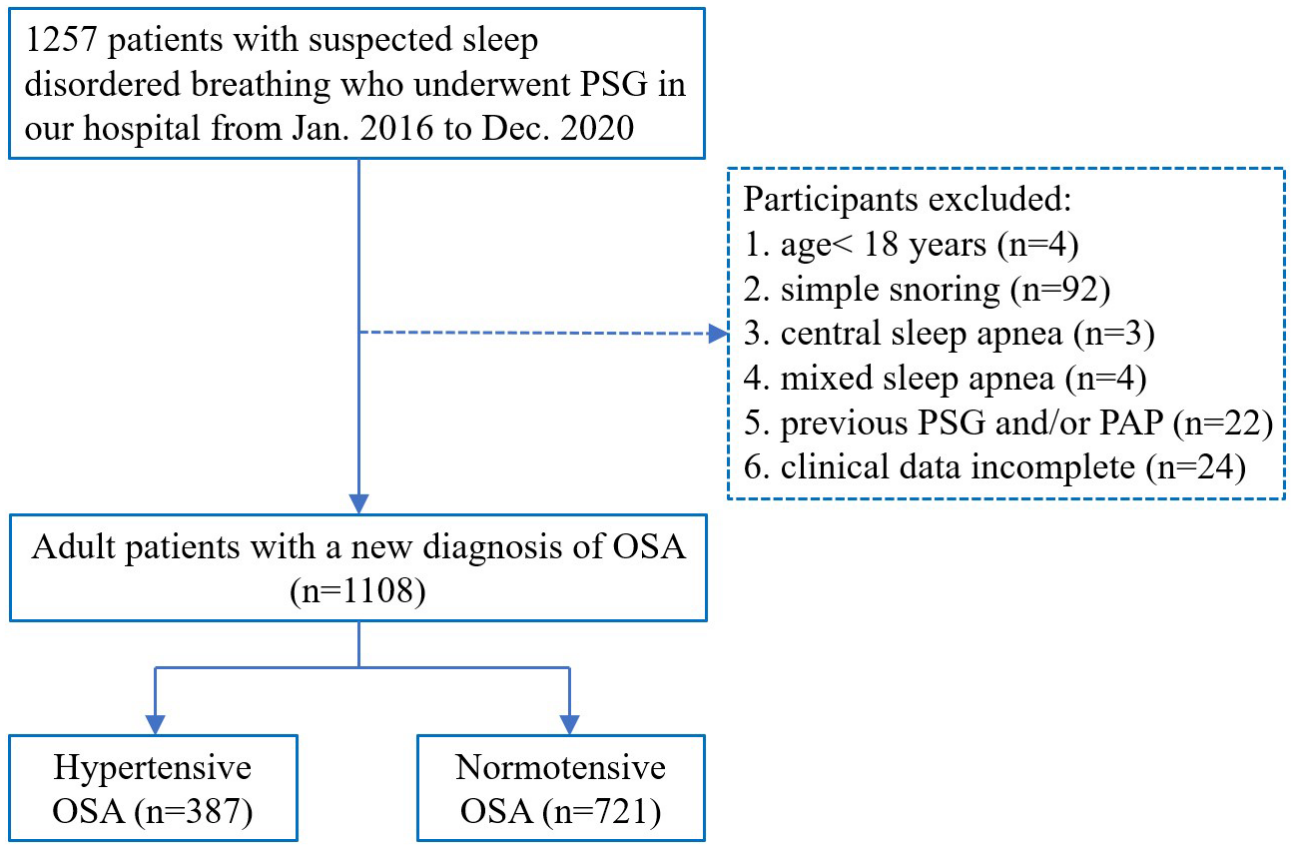
Flow chart of the patients’ inclusion process. PSG, polysomnography; OSA, obstructive sleep apnea; PAP, positive airway pressure.

This retrospective study used data from January 2016 to December 2020; 1257 patients who were admitted to the affiliated Lihuili Hospital of Ningbo University with suspected sleep disordered breathing underwent full-night PSG examination, and adult patients with newly diagnosed OSA were included in this study. The exclusion criteria were age<18 y, simple snoring, central sleep apnea, mixed sleep apnea, previous PSG and/or continuous positive airway pressure treatment, and incomplete clinical data. A total of 1108 patients met the inclusion criteria, including 387 with hypertension and 721 without hypertension. Hypertension in this study was defined according to Chinese Guidelines for the Prevention and Treatment of Hypertension: In the absence of antihypertensive drugs, room blood pressure was measured three times on different days, SBP≥140mmHg and/or DBP≥90 mmHg or the patient’s history of a definite diagnosis of hypertension. The grouping flow chart is presented in **Figure 1**. The study was approved by the Ethics Committee of The Affiliated Lihuili Hospital of Ningbo University (Approval Number: KY2021PJ241) and written informed consent was obtained from each participant.

### 2.2 Data collection

Age and sex of the participants were obtained from an inpatient system. Professional nurses measured the height, weight, and awake pulse oxygen saturation of the participants. Detailed medical histories of the participants, including comorbidities and other diseases, were collected and recorded by professional respiratory physicians. Daytime sleepiness was assessed using the Epworth Sleepiness Scale (ESS). Body mass index (BMI) was defined as kg/m^2^.

### 2.3 Polysomnography

Participants underwent full-night PSG monitoring in the hospital’s sleep-monitoring room and were asked to avoid alcohol, tea, coffee, sedation, and hypnotic drugs that day. We had used a polysomnographic system (Alice 5, Respironics, Philips, Pittsburgh, Pennsylvania, USA) to test at least 7 hours of sleep. The monitoring items included electroencephalography, electrocardiography, electroophthalmography, mandibular electromyography, oronasal airflow, snoring, chest and abdominal movements, body position, and pulse oxygen saturation.

### 2.4 Data analysis

Continuous variables which conform to normal distribution are expressed by mean and standard deviation, those that do not conform to normal distribution are expressed by median (quartile), and categorical variable data are expressed by percentage. The t-test or analysis of variance (ANOVA) were used to compare continuous variables, and the chi-squared test was used to compare categorical variables. Multivariate analysis was performed using binary logistic regression. SPSS26.0 statistical analysis software was used for statistical analysis, and a P value <0.05 was considered statistically significant.

## 3 Results

### 3.1 Sociodemographic characteristics

The sociodemographic characteristics of the 1108 patients with OSA are shown in **Table 1**. There were 387 patients with hypertension and 721 patients without hypertension, respectively. The average age of the total patients was 45.30 ± 11.58 y; the average age of patients with and without hypertension was 50.31 ± 10.92 and 42.6 ± 11.03 y, respectively. The mean age of patients with hypertension was significantly higher than that of patients without hypertension (P < 0.001). There was a significant increase in BMI (P < 0.001) and ESS scores (P =0.048) in patients with hypertension. The proportions of obese patients (P < 0.001) and smokers (P =0.003) with hypertension were significantly higher. No differences in sex or alcohol use were observed between OSA patients with and without hypertension.

**Table 1:** Sociodemographic characteristics of total OSA patients and the patients with or without hypertension.

	Total (n=1108)	With hypertension (n=387)	Without hypertension (n=721)	T/F/X^2^	P-value
Age (years)	45.30 ± 11.58	50.31 ± 10.92	42.6 ± 11.03	-11.13	<0.001
Male, n (%)	936 (84.48)	316 (81.65)	620 (85.99)	3.61	0.057
Female, n (%)	172 (15.52)	71 (18.35)	101 (14.01)		
BMI (kg/m²)	27.19 ± 3.41	28.25 ± 3.56	26.62 ± 3.18	-7.76	<0.001
Obese patients, n (%)	416 (37.55)	195 (50.39)	221 (30.65)	41.83	<0.001
ESS score	8.00 (4.00 - 12.00)	8.00 (5.00 - 12.00)	7.00 (4.00 - 11.00)	-1.97	0.048
Smoking, n (%)	164 (14.80)	74 (19.12)	90 (12.48)	8.80	0.003
Alcohol use, n (%)	84 (7.58)	36 (9.30)	48 (6.66)	2.51	0.113

BMI, body mass index; ESS, Epworth Sleepiness Scale.

### 3.2 Clinical symptoms

**Table 2** shows the clinical symptoms of total patients with OSA and those with or without hypertension. The proportions of patients waking up at night (P =0.015), mouth dryness (P=0.010), nocturia (P =0.010), memory deterioration(P<0.010), and impatient personality (P=0.010) were significantly increased in OSA patients with hypertension. There were no differences in the ratio of morning headache, daytime sleepiness, impact on quality of life, work, or traffic safety.

**Table 2:** Clinical features of total OSA patients and the patients with or without hypertension.

	Total (n=1108)	With hypertension (n=387)	Without hypertension (n=721)	X^2^	P-value
Waking up at night, n (%)	427 (38.54)	168 (43.41)	259 (35.92)	5.96	0.015
Morning headache, n (%)	192 (17.33)	72 (18.60)	120 (16.64)	0.68	0.411
Mouth Dryness, n (%)	714 (64.44)	269 (69.51)	445 (61.72)	6.67	0.010
Daytime sleepiness, n (%)	588 (53.07)	220 (56.84)	368 (51.04)	3.41	0.065
Nocturia, n (%)	309 (27.89)	159 (41.09)	150 (20.80)	51.51	<0.001
Memory deterioration, n (%)	675 (60.92)	267 (68.99)	408 (56.59)	16.28	<0.001
Impatient personality, n (%)	392 (35.38)	161 (41.60)	231 (32.04)	10.07	0.002
Impact of life quality, n (%)	431 (38.90)	151 (39.02)	280 (38.84)	0.004	0.952
Impact of work, n (%)	304 (27.44)	107 (27.65)	197 (27.32)	0.01	0.908
Impact of traffic safety, n (%)	167 (15.07)	61 (15.76)	106 (14.70)	0.22	0.638

### 3.3 Clinical comorbidities

Other clinical comorbidities are shown in **Table 3**. The proportions for diabetes mellitus (P <0.010), coronary heart disease (P <0.010), chronic obstructive pulmonary disease (P =0.045), and ischemic stroke (P <0.010) were significantly higher in OSA patients with hypertension. There were no statistically significant differences in the proportions of gastroesophageal reflux and thyroid disease between OSA with and without hypertension.

**Table 3:** Clinical comorbidities of total OSA patients and the patients with or without hypertension.

	Total (n=1108)	With hypertension (n=387)	Without hypertension (n=721)	X^2^	P-value
Diabetes mellitus, n (%)	95 (8.60)	68 (17.57)	27 (3.74)	61.41	<0.001
Cardiovascular disease, n (%)	95 (8.57)	58 (14.99)	37 (5.13)	31.20	<0.001
Chronic obstructive pulmonary disease, n (%)	45 (4.06)	22 (5.68)	23 (3.19)	4.02	0.045
Ischemic stroke, n (%)	27 (2.44)	6 (1.61)	21 (2.85)	22.36	<0.001
Gastroesophageal reflux, n (%)	119 (10.74)	50 (12.92)	69 (9.57)	2.95	0.086
Thyroid disease, n (%)	36 (3.25)	12 (3.10)	24 (3.33)	0.04	0.838

### 3.4 PSG results

**Table 4** shows the PSG results of all patients with OSA and those with or without hypertension. Awake oxygen saturation (P <0.010), minimum oxygen saturation (P <0.010), and average oxygen saturation (P <0.010) significantly decreased in OSA patients with hypertension. The sleep latency (P =0.008), N1 ratio (P=0.010), N3 ratio (P=0.010), AHI (P <0.010), oxygen desaturation index (P <0.010), and percentage of recording time with SaO_2_ <90% (T90) (P <0.010) differed significantly between hypertensive and normotensive OSA patients. The results of other PSG tests were not statistically significant.

**Table 4:** PSG result of total OSA patients and the patients with or without hypertension.

	Total (n=1108)	With hypertension (n=387)	Without hypertension (n=721)	T/F/X^2^	P-value
Awake oxygen saturation (%)	96.75 ± 0.95	96.58 ± 0.93	96.84 ± 0.96	4.73	<0.001
Sleep efficiency (%)	88.85 (79.40 - 94.10)	88.80 (79.00 - 94.30)	88.90 (79.70 - 93.90)	-0.66	0.510
Wake after sleep onset (minutes)	27.50 (12.50 - 65.38)	26.50 (12.50 - 64.50)	28.00 (13.00 - 66.00)	-0.23	0.821
Sleep latency (minutes)	19.00 (10.50 - 35.38)	17.00 (9.50 - 32.00)	20.00 (11.00-36.50)	-2.67	0.008
N1 ratio (%)	38.40 (27.60 - 52.70)	41.10 (30.60 - 55.70)	36.90 (26.60 - 52.10)	-3.34	0.001
N2 ratio (%)	45.40 (34.55 - 55.40)	44.60 (34.10 - 53.80)	45.90 (34.85 - 56.00)	-1.57	0.116
N3 ratio (%)	2.10 (0.00 - 9.40)	0.90 (0.00 - 7.90)	2.90 (0.00 - 10.25)	-3.35	0.001
R ratio (%)	7.20 (1.00 - 12.60)	6.70 (0.60 - 11.60)	7.40 (1.30 - 12.95)	-1.55	0.122
Microarousal index (times/hour)	45.00 (29.10 - 61.30)	46.10 (29.80 - 62.70)	43.80 (28.70 - 58.60)	-1.51	0.131
AHI (times/hour)	41.65 (21.13 - 62.27)	48.20 (28.20 - 65.90)	37.30 (16.35 - 60.40)	-4.69	<0.001
Mild OSA, n (%)	210 (18.95)	47 (12.14)	163 (22.61)	27.11	<0.001
Moderate OSA, n (%)	195 (17.60)	56 (14.47)	139 (19.28)		
Severe OSA, n (%)	703 (63.45)	284 (73.39)	419 (58.11)		
Maximum duration of apnea (seconds)	55.50 (36.00)	57.50 (39.50-74.50)	55.00 (38.00-74.50)	-0.72	0.472
Minimum oxygen saturation (%)	72.23 ± 13.04	70.19 ± 13.84	73.33 ± 12.46	3.85	<0.001
Average oxygen saturation (%)	92.86 ± 3.39	92.27 ± 3.80	93.18 ± 3.10	4.06	<0.001
ODI (times/hour)	40.90 (20.83 - 63.38)	47.60 (27.50 - 67.30)	36.10 (16.90 - 61.15)	-5.28	<0.001
T90 (%)	8.07 (1.44 - 22.99)	11.50 (2.27 - 26.36)	6.30(1.10 - 20.75)	-3.99	<0.001

AHI, apnea-hypopnea index; ODI, oxygen desaturation index; T90, percentage of recording time with SaO_2_ <90%.

### 3.5 Multi-variate analysis

When controlling for sociodemographic features, clinical symptoms, comorbidities, and PSG results (**Table 5**), binary logistic regression analysis suggested that age, BMI, ESS score, nocturia, diabetes, cardiovascular disease, and ischemic stroke were independently associated with hypertension in OSA patients.

**Table 5:** Independent risk factors of OSA patients with hypertension.

	B-value	Standard error	P-value	OR-value	95% CI for OR
Age (years)	0.06	0.01	<0.001	1.06	1.04 - 1.08
BMI (kg/m²)	0.15	0.03	<0.001	1.17	1.11 - 1.23
ESS score	-0.03	0.02	0.047	0.97	0.94 - 1.00
Smoking, n (%)	0.32	0.20	0.116	1.38	0.92 - 2.06
Waking up at night, n (%)	-0.05	0.15	0.744	0.95	0.70 - 1.28
Dry, n (%)	0.12	0.16	0.469	1.12	0.82 - 1.54
Nocturia, n (%)	0.50	0.16	0.002	1.64	1.19 - 2.27
Memory deterioration, n (%)	0.31	0.16	0.056	1.36	0.99 - 1.86
Impatient personality, n (%)	0.18	0.16	0.246	1.20	0.88 - 1.64
Diabetes, n (%)	1.35	0.26	<0.001	3.86	2.31 - 6.45
Cardiovascular disease, n (%)	0.64	0.26	0.013	1.90	1.15 - 3.16
Chronic obstructive pulmonary disease, n (%)	-0.36	0.36	0.312	0.70	0.35 - 1.40
Ischemic stroke, n (%)	1.31	0.53	0.014	3.69	1.31 - 10.40
Awake oxygen saturation (%)	0.05	0.09	0.558	1.05	0.89 - 1.24
Sleep latency (minutes)	0.00	0.00	0.415	1.00	0.99 - 1.00
N1 ratio (%)	0.00	0.00	0.819	1.00	0.99 - 1.01
N3 ratio (%)	-0.01	0.01	0.251	0.99	0.98 - 1.01
AHI (times/hour)	0.00	0.01	0.882	1.00	0.98 - 1.02
Minimum oxygen saturation (%)	0.00	0.01	0.859	1.00	0.98 - 1.02
Average oxygen saturation (%)	0.05	0.04	0.231	1.05	0.97 - 1.13
ODI (times/hour)	0.01	0.01	0.491	1.01	0.99 - 1.03
T90 (%)	0.01	0.00	0.159	1.01	1.00 - 1.02

B-value, log-odds; OR, odds ratio; BMI, body mass index; ESS, Epworth Sleepiness Scale; AHI, apnea–hypopnea Index; ODI, oxygen desaturation index; T90, percentage of recording time with SaO_2_ <90

## 4 Discussion

OSA is a complex and heterogeneous disease with multiple pathophysiological results such as chronic intermittent hypoxia, oxidative stress, inflammatory response, increased negative pressure in the chest, and increased sympathetic activity (2). Peppard et al. found a dose-effect relationship between baseline sleep-disordered breathing and hypertension after four years, independent of known confounding factors (20). This suggests that the risk of developing hypertension increases with AHI. Sleep in patients with OSA is highly fragmented, with a higher proportion of light sleep; the proportion of slow wave sleep (SWS) is significantly decreased, and SWS stage is accompanied by lower sympathetic nervous activity and higher parasympathetic nerve activity (3, 21). Experimental inhibition of human SWS can significantly reduce the drop in blood pressure at night, that is, the increase in blood pressure at night; a decrease in the proportion of SWS is considered a sign of elevated blood pressure (22). In patients with OSA, blood pressure is characterized by no decrease in blood pressure, nocturnal hypertension, and increased blood pressure variability, that is, non-dipping or rising patterns (23–27). Elevated nighttime BP and higher morning BP are associated with increased risk of cardiovascular events and worse cardiovascular outcomes (28, 29). In our study, SWS was statistically significant in the univariate analysis, but not in the multivariate analysis, which may be related to the imbalance of other factors, such as age, between the two groups. Through meta-analysis, Maurice et al. found that sleep time, sleep efficiency, and SWS decrease with increasing age (30).

The most commonly used quantitative method to assess the degree of daytime sleepiness is the subjective ESS. When the ESS score was greater than 10 was defined as EDS (31). EDS has become one of the most common and prominent symptoms in patients with OSA and is an important criterion for its diagnosis and treatment (32, 33). In this study, we found that the ESS score of OSA patients with hypertension was significantly higher than that of those without hypertension. Kapur et al. suggested that participants with sleep-disordered breathing who often felt excessively sleepy were more likely to develop high blood pressure than those who did not (34). Feng et al. found that OSA patients with higher ESS scores were more likely to have higher blood pressure than OSA patients with lower ESS scores, and even had a higher risk of being diagnosed with hypertension (35). Robinson et al. reported that EDS hypertensive patients with OSA experienced a decrease in blood pressure after receiving continuous positive airway pressure (CPAP) compared without EDS hypertensive patients (36). This suggests that daytime sleepiness may be related in some way to the pathogenesis of hypertension in sleep apnea. However, contrary results have also been reported in the literature. Martynowicz et al. found that in the moderate to severe OSA group, the total ESS score of hypertensive patients was significantly lower than that of patients with normal blood pressure (37). This may be because the ESS relies on patient self-reporting, and is susceptible to subjective factors.

Nocturia refers to the occurrence of two or more urinations at night (38). The main causes of nocturia include inflammation, benign prostatic hyperplasia, excessive bladder activity, neurogenic bladder, and OSA. Nocturia and nighttime hypertension are common in patients with obstructive sleep apnea (OSA) and share several physiopathological pathways (27). AHI is an independent predictor of nocturnal urination frequency in OSA patients (39). Nocturia became more common as the severity of OSA increased. OSA may lead to nocturia by several mechanisms: OSA can lead to airflow obstruction, intermittent hypoxia, sympathetic hyperactivity, and changes in thoracic pressure, resulting in atrial and ventricular secretion of atrial natriuretic peptide (ANP) and brain natriuretic peptide (BNP), stimulating water and sodium excretion, inhibiting RAAS and antidiuretic hormone (ADH) release (40–42). Consistent with our findings, Destors et al. showed that nocturia was strongly associated with OSA-associated hypertension after adjusting for confounders (43). Rahman et al. found that compared with patients with normal blood pressure, patients with hypertension had a 1.2-1.3 times increased risk of nocturia (44). Although the pathophysiological mechanism of the association between hypertension and nocturia has not been fully established, several hypotheses have been proposed. First, higher daytime blood pressure may reflect higher nighttime blood pressure, which promotes natriuresis (45). Hypertension can lead to nocturia through changes in glomerular filtration rate and tubular transport (46). Nocturnal polyuria due to RAAS activation, a corresponding increase in nocturnal sodium in the urine, and decreased bladder function due to chronic vascular insufficiency are mediated by hypertension and other atherosclerotic risk factors (47). Congestive heart failure due to hypertension leads to atrial extension, increased release of atrial natriuretic peptide (ANP) and increased nocturia due to renal hyperfiltration (48). Therefore, nocturia was more frequent in OSA patients with hypertension than in those without hypertension.

Diabetes is a common comorbidity of OSA, and increasing evidence supports a complex relationship between OSA and diabetes. Intermittent hypoxemia and sleep disruption can lead to activation of the sympathetic nervous system, increased oxidative stress, dysregulation of the hypothalamic-pituitary-adrenal (HPA) axis, and systemic inflammation. Sympathetic activation leads to increased levels of catecholamines such as norepinephrine and epinephrine, inducing insulin resistance, inhibiting pancreatic insulin secretion, and resulting in elevated blood sugar (49). Adrenaline can also promote hepatic glucose production and inhibit insulin secretion, and the uptake of glucose by tissues further increases blood glucose uptake (50). OSA can lead to a significant reduction or inhibition of SWS (21), and Herzog et al. found that selective suppression of SWS resulted in an increase in plasma glucose and a decrease in insulin sensitivity in the early morning (51). However, several studies have found that the relationship between OSA and type 2 diabetes may be bidirectional, and that diabetic neuropathy can affect central respiratory control and the upper respiratory tract nerve reflex and promote sleep-disordered breathing (52). Diabetes and hypertension have overlapping genetic, physiological, and environmental factors, and hypertension may be considered as an important cause of diabetes (53, 54). Diabetic complications are more common in patients with OSA and hypertension than in those without. Luo et al. found that non-falling blood pressure in hypertensive patients with OSA was associated with an approximately 1.5-fold increased risk of new-onset diabetes (55). This suggests that hypertension in OSA may induce or promote diabetes.

OSA is closely associated with cardiovascular and cerebrovascular diseases including transient ischemic attack, stroke, pulmonary hypertension, heart failure, coronary heart disease, atrial fibrillation, myocardial infarction, and sudden death (56). Intermittent hypoxia, systemic inflammation, sympathetic nerve activation, and endothelial dysfunction caused by OSA are important mechanisms that induce cardiovascular and cerebrovascular diseases (56–59). The strong association between OSA and coronary and cerebrovascular disease appears to be independent of shared risk factors, including obesity (60). Most studies have shown a causal relationship between OSA severity (as assessed using AHI) and the risk of cardiovascular events (61). It has also been found that the risk of stroke in patients with mild OSA is 2.44 times higher than that in normal people, while the risk of stroke in patients with moderate or severe OSA is 3.56 times higher than that in normal people (62). This indicates that the severer OSA is, the more likely it is to be complicated with cardiovascular and cerebrovascular diseases. Previous studies have found that the relationship between OSA and cardiovascular disease risk factors is bidirectional, suggesting that patients with cardiovascular disease may also develop OSA and that effective treatment of cardiovascular disease may improve OSA (63). Compared with patients without hypertension, hypertension also significantly increases the risk and severity of cardiovascular and cerebrovascular diseases (64). High blood pressure is an intermediary between OSA and cardiovascular disease, and its presence of high blood pressure also increases the risk of cardiovascular development in patients with OSA, ultimately increasing the risk of cardiovascular-related death (65). In addition, OSA has been shown to be an independent risk factor for hypertension, ischemic heart disease, and arrhythmia, which in turn constitute risk factors for ischemic stroke (66). Therefore, hypertensive OSA is associated with a significantly increased risk for cardiovascular and cerebrovascular diseases.

The advantage of this study is that the sample size was large, covering all genders and age stages, and the sample data were rich, including sociodemographic features, clinical characteristics, and PSG results. However, this study also had some limitations. First, ambulatory blood pressure monitoring was not performed in this study and data of blood pressure at night were unavailable. Second, there was no subgroup analysis of hypertension level.

## 5 Conclusion

In conclusion, our study found that patients with OSA and hypertension had more severe daytime sleepiness and more frequent nocturnal urination than OSA patients without hypertension. Simultaneously, there is a higher risk of combining with diabetes, coronary heart disease, and ischemic stroke. This indicates that the patients with OSA combined with hypertension had more symptoms and a heavier burden of cardiovascular, and cerebrovascular, and metabolic complications. More attention should be paid to these patients, and individualized treatment should be provided in clinical practice.

## Data availability statement

The original contributions presented in the study are included in the article/supplementary material. Further inquiries can be directed to the corresponding author.

## Ethics statement

The studies involving humans were approved by the Ethics Committee of The Affiliated Lihuili Hospital of Ningbo University. The studies were conducted in accordance with the local legislation and institutional requirements. The participants provided their written informed consent to participate in this study.

## Author contributions

MT: Formal Analysis, Methodology, Writing – original draft, Writing – review & editing. XD: Resources, Writing – review & editing. JT: Resources, Writing – review & editing. QF: Resources, Writing – review & editing. CS: Conceptualization, Funding acquisition, Investigation, Methodology, Project administration, Resources, Supervision, Validation, Writing – original draft, Writing – review & editing.
